# Primary malignant melanoma of the lung: a case report and literature review

**DOI:** 10.1186/s12890-020-1140-z

**Published:** 2020-04-17

**Authors:** Shuangshuang Deng, Xiaobo Sun, Zhen Zhu, Jingjing Lu, Guanghua Wen, Xuejiao Chang, Hui Gao, Yanfei Hua, Lumei Wang, Jinli Gao

**Affiliations:** 1 Department of Pathology Medicine, Shanghai East Hospital, Tongji University School of Medicine, #150 Jimo Road, Pudong, 200120 Shanghai China; 2 Department of Respiratory Medicine, Shanghai East Hospital, Tongji University School of Medicine, Shanghai, 200120 China; 3 Department of Nuclear Medicine, Jinhua Central Hospital, Jinhua, 321000 Zhejiang China

**Keywords:** Malignant melanoma, Diagnosis, Primary malignant melanoma of the lung (PMML), Lung cancer, Pathology

## Abstract

**Background:**

Malignant melanoma (MM) generally presents as a primary neoplasm of the skin, and most MM cases of the respiratory system are metastatic. Primary MM of the lung (PMML) is quite rare, and its diagnosis is relatively difficult.

**Case presentation:**

We report the case of a 57-year-old male patient with PMML who denied any history of tumours. His initial complaint was frequent coughs with bloody sputum for 4 days. Chest radiography demonstrated a high-density shadow in the lower lobe of the right lung, which was suspected to be a large space-occupying lesion on subsequent computed tomography (CT) and to be a hypermetabolic tumour by positron emission tomography–CT. To confirm the diagnosis, exploratory surgery was performed. Finally, we confirmed the diagnosis of PMML.

**Conclusions:**

PMML is extremely rare and easily misdiagnosed as lung cancer. Because of its morphological and immunophenotypic variations, the diagnosis of PMML remains difficult. This case report discusses the diagnosis and case management of a patient while referring to the existing literature.

## Background

Malignant melanoma (MM) is a refractory malignant tumour. In 2015, approximately 351,880 new cases of MM were diagnosed worldwide [1]. MM is very aggressive and can metastasise in an early phase of the disease. MM generally presents as a primary neoplasm of the skin but may also arise in other organs and tissues, such as the respiratory tract, oral cavity, liver, ovaries, oesophagus, larynx, cervix, vagina and gallbladder. Most MM cases of the respiratory system are metastatic at the time of diagnosis, and primary malignant melanoma of the lung (PMML) is quite rare, accounting for only 0.01% of all primary lung tumours and 0.4% of all MMs [2]. In this article, we report the case of a 57-year-old PMML patient with a small solid nodule of the lung on initial computed tomography (CT), which was later confirmed as PMML by postoperative pathology. In addition, we discuss the clinicopathological features of PMML by reviewing the relevant literature.

## Case presentation

**Fig. 1:**
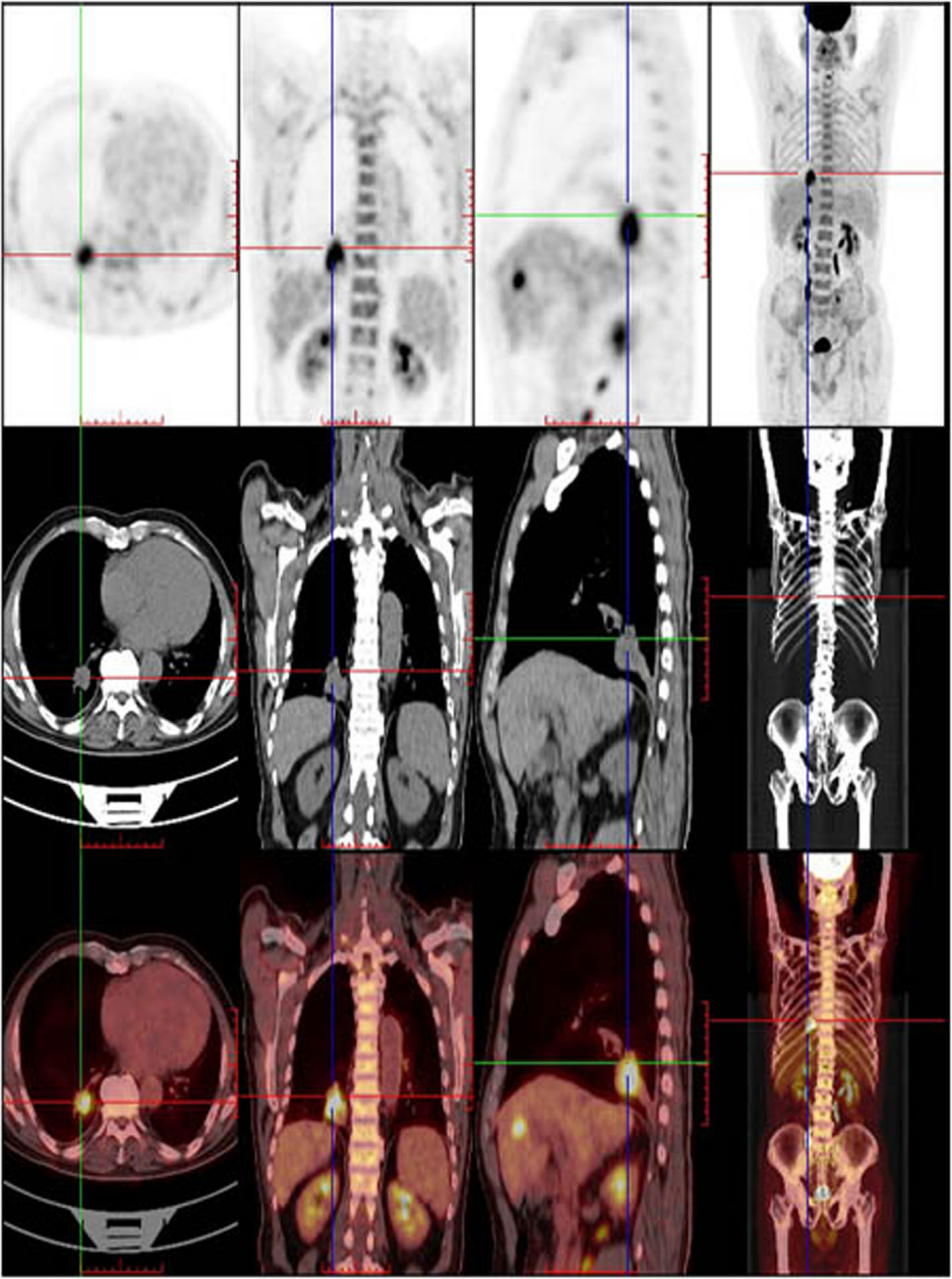
PET-CT revealed a malignant space-occupying lesion in the lower lobe of the right lung. CT, computed tomography; PET, positron emission tomography

A 57-year-old man presented to Jinhua Central Hospital affiliated with Zhejiang University (Jinhua, China) with a complaint of frequent cough with bloody sputum for 4 days. He denied any history of skin, mucous membrane or eye surgeries; electric cauterisation; or any family history of cancer. Physical examination at the outpatient clinic revealed diminished breath sounds over the lower lobe of the right lung, and no abnormal lesions were detected in other sites of the body, including the skin, head, neck, scalp, anogenital region and eyes. Chest radiography showed a high-density shadow. Subsequent CT plain and contrast-enhanced scan showed a space-occupying lesion in the lower lobe of the right lung adjacent to the pleura with a clear boundary. A right lower lobectomy for the space-occupying lesion was performed and further diagnosed by pathological examination. Before the excision, whole body positron emission tomography–CT (PET-CT) was performed, showing a malignant space-occupying lesion in the lower lobe of the right lung with liver metastasis (Fig. 1).

**Fig. 2:**
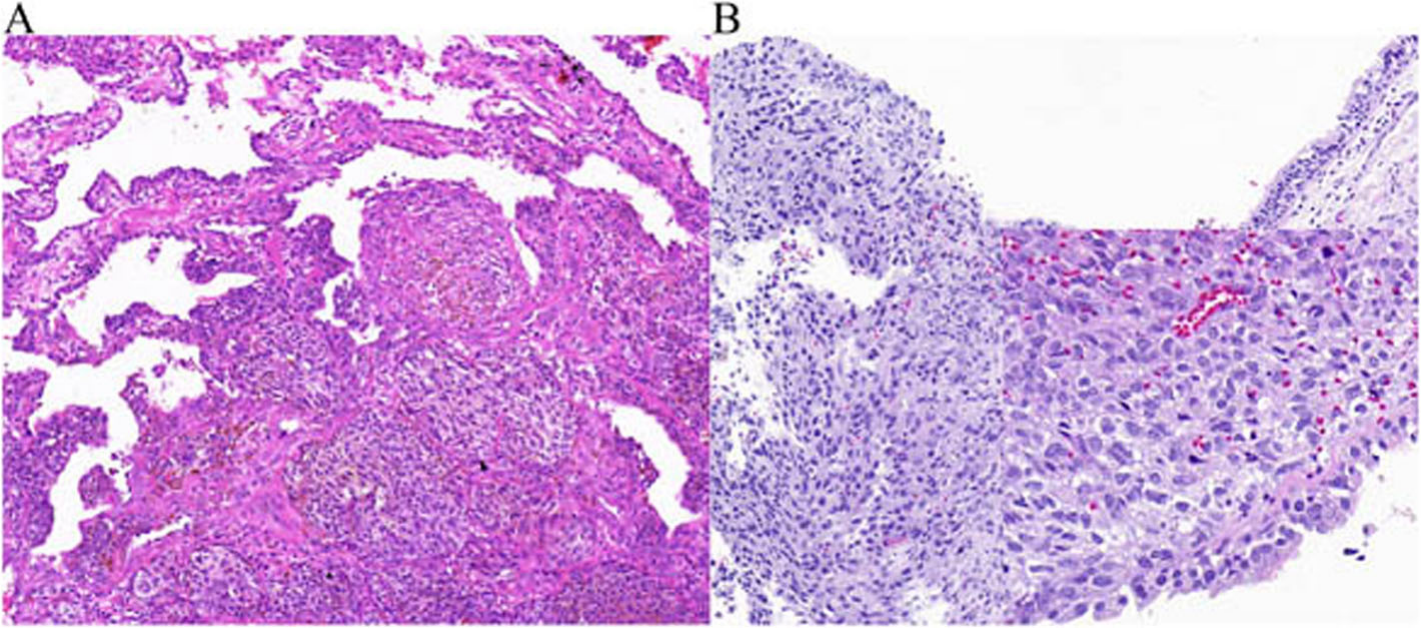
Hematoxylin and eosin staining. **a** The tumour comprised malignant tumour cells with large amounts of acidophilic cytoplasm. Melanin pigmentation could also sometimes be noticed. **b** Tumour cells invaded the bronchial subepithelial area from the basement membrane

**Fig. 3:**
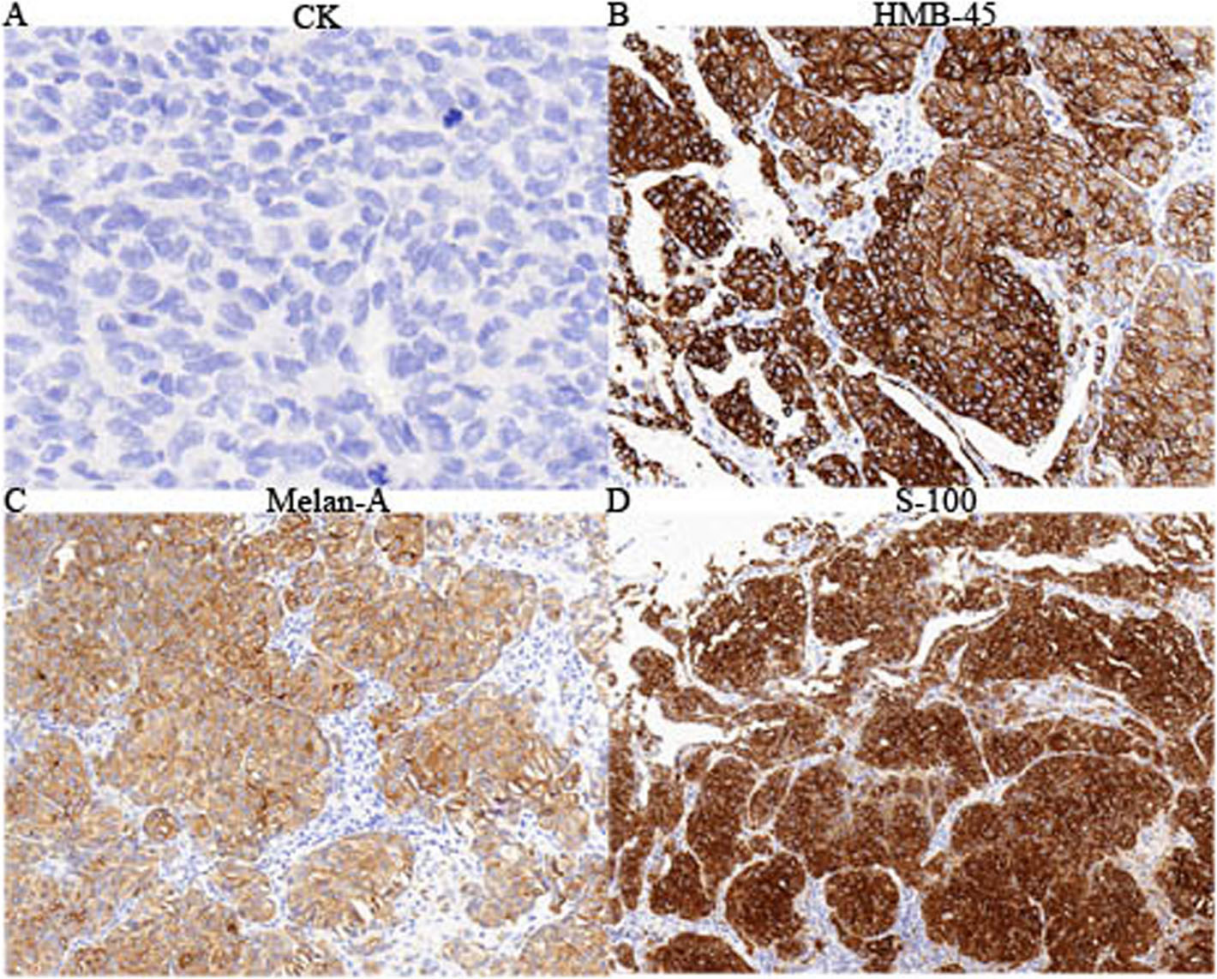
Immunohistochemical staining (400×). The cytoplasm of the malignant cells stained negative for CK (**a**) and strongly positive with antibodies against HMB-45 (**b**), S-100 (**c**), and Melan-A (**d**)

Grossly, a solitary mass measuring 3.5 cm × 3.0 cm × 3.0 cm was located in the lower lobe of the right lung, appearing as a round, darkly pigmented, solid neoplasm with significant necrosis. Hematoxylin and eosin staining demonstrated that the tumour was located in the lung tissue, comprising malignant epithelial tumour cells with large amounts of acidophilic cytoplasm and prominent nuclei. Melanin pigmentation could also sometimes be noticed, and there were junctional changes with characteristic tumour cells invading the bronchial subepithelial area from the basement membrane. All of these findings were suggestive of MM (Fig. 2). Immunohistochemical staining demonstrated that the cytoplasm of the malignant cells was positive for human melanoma black 45 (HMB-45), Melan-A, and S-100 and negative for cytokeratin (CK), CK7, Napsin A, transcription termination factor 1 (TTF1), P40 and P63, confirming the diagnosis of MM (Fig. 3).

Based on the clinical characteristics, CT and PET-CT findings and pathological evaluation, a final diagnosis of PMML was made. After surgery, the patient received one cycle of chemotherapy. However, as his general condition was very poor, the patient died of pulmonary infection 27 days later.

## Discussion and conclusions

PMML is extremely rare but characterised by high malignancy, a high recurrence rate and poor prognosis. Only about 47 cases have been reported in the literature [3, 4]. The pathogenesis of PMML remains controversial. There are several theories to explain its occurrence [5–7]: (1) melanocytes exist in the whole body as cells of a dispersed neuroendocrine system; (2) melanocytes migrate to the respiratory tract during embryogenesis, where they transform into malignant cancer cells; (3) melanocytes may derive from melanoblasts and produce PMML because they have the same origin as other melanoblasts located in the trachea, oesophagus, and pharynx [8–10]; and (4) melanoma cells may originate from pluripotent stem cells. More research is required to clarify the pathogenesis of PMML.

The diagnosis criteria of PMML have been clearly defined by Jensen and Egedorf [11], with only minor modifications, including the clinical characteristics, radiologic features, and pathological diagnosis [12]. The prerequisite of the clinical characteristics includes no previously resected pigmented skin lesion, absence of any other detectable tumours at the time of diagnosis, no history of a melanoma in other organs at time of surgery, and solitary lung cancer. The prerequisite of the radiologic features includes an abnormal shadow on chest radiography and an irregular mass or node on CT. The prerequisite of pathological diagnosis includes tumour morphology comparable with that of MM, invasion of the bronchial epithelium by melanoma cells, junctional changes including ‘dropping off’ or ‘nesting’ beneath the bronchial epithelium, and melanoma often accompanied by complex morphological variations [13], including (1) histological structural variations as typically represented by cell arrangement in a nest, aciniform, trabecular, mamillary, swirling, pseudorosettes, or glomeruli shape; (2)cytological variations as typically represented by the presence of giant, large-sized, medium-sized, small-sized, spindle, signet ring–like, rhabdomyoblast-like, plasmacytoid cells, or balloon-like cells; and (3) varying degrees of interstitial changes such as fibrogenesis, mucoid degeneration and inflammatory cell infiltration. The pathological diagnosis of melanoma is sometimes difficult because of these variations. Based on radiography, CT and PET-CT findings; clinical characteristics, and pathological diagnosis, the present case fulfilled the above-mentioned diagnostic criteria and was compatible with a diagnosis of PMML. In the present case, HMB-45 and Melan-A staining showed a diffuse strong positivity, which is valuable for the diagnosis of MM, considering that HMB-45 is a very specific marker of primary melanoma [8, 9, 14] and Melan-A has high sensitivity and specificity for the diagnosis of MM. In addition, PET-CT results showed no evidence of malignancy in other sites, except for the liver and lung, and a complete examination of the skin and ocular tumours showed no melanocytic lesion. Thus, we diagnosed the lung tumour as PMML.

During the diagnosis, attention should be paid to distinguishing PMML from some similar tumours such as squamous cell carcinoma (SCC), pulmonary metastatic melanoma (PMM), and pigmented carcinoid tumour (PCT) [15–17]. The imaging features of primary MM are similar to those of SCC, especially when the degree of tumour differentiation is low. Histologically, it is difficult to distinguish PMML from lung SCC, and therefore, it is important to know that immunohistochemistry staining of SCC is usually positive for CKpan, P63 and P40 but negative for S-100, HMB-45 and Melan-A. PMM usually has multiple nodules or masses that can be detected in the lung, whereas primary melanoma often appears as a solitary nodule or mass, often involving the bronchial lumen. Although PCT and PMML have very similar morphology, PCT often presents argyrophilic or argentophil granules. In addition, immunohistochemistry staining often shows that PCT is positive for CK, CD56, synaptophysin, chromogranin-A and neuroendocrine granules can be observed under the electron microscope.

The clinical presentation of PMML varies with individual cases. Yamamoto et al. [18] reported a case of PMML in a 61-year-old female patient who died of the disease 15 months after the operation. Yabuki et al. [19] reported a similar case in a 74-year-old male patient with PMML, who died 7 months after right lower lobectomy. Most PMML patients have a very short survival time and die within months of diagnosis. However, Madeline et al. [20] reported a case of PMML in a patient who remained disease-free 60 months after pneumonectomy. In addition, younger PMML survivors were rarely seen. Recently, Yunce et al. [21] reported a resection in a 22-year-old Caucasian. In our case, the PMML patient was a 57-year-old who succumbed to the disease 1 month after operation. In all, most PMML patients were of older age with diverse clinical presentations, and the prognosis was usually poor.

In summary, PMML is extremely rare and easily misdiagnosed as lung cancer. Histopathological examination is a reliable gold standard for confirmation of a PMML diagnosis. Because of the morphological and immunophenotypic variations, the diagnosis of PMML remains difficult and should be distinguished from the diagnosis of other cancers. Immunohistochemistry staining is helpful for the differential diagnosis of PMML. Because of the lack of clinical data and clear understanding of the mechanisms underlying the pathogenesis of PMML, there are no consensus guidelines on the treatment of PMML. We hope that the case reported here will add more clinical data to the current information about PMML and help to provide more constructive suggestions about the treatment of PMML.

## Data Availability

The dataset supporting the findings and conclusions of this case report is included within the article and Figures.

## Abbreviations

PMML: Primary malignant melanoma of the lung
MM: Malignant melanoma
SOL: Space-occupying lesion
RLL: A right lower lobectomy
PMM: Pulmonary metastatic melanoma
SCC: Squamous cell carcinoma
PCT: Pigmented carcinoid tumor
CT: Computed tomography
PET-CT: Positron emission tomography-computed tomography
HMB-45: Human melanoma black
TTF1: Transcription termination factor 1
PMM: Pulmonary metastatic melanoma
SCC: Squamous cell carcinoma
SYN: Synaptophysin
CgA: Chromogranin-A

## References

[1] Karimkhani C, Green AC, Nijsten T (2017). The global burden of melanoma: results from the global burden of disease study 2015. Br J Dermatol.

[2] Wilson RW, Moran CA (1997). Primary melanoma of the lung: a clinicopathologic and immunohistochemical study of eight cases. Am J Surg Pathol.

[3] Testini M, Trabucco S, Di Venere B, Piscitelli D (2002). Ileal intussusception due to intestinal metastases from primary malignant melanoma of the lung. Am Surg.

[4] Gong L, Liu XY, Zhang WD, Zhu SJ, Yao L, Han XJ (2012). Primary pulmonary malignant melanoma: a clinicopathologic study of two cases. Diagn Pathol.

[5] Shi Y, Bing Z, Xu X, Cui Y (2018). Primary pulmonary malignant melanoma: case report and literature review. Thorac Cancer.

[6] Peng J, Han F, Yang T, Sun J, Guan W, Guo X (2017). Primary malignant melanoma of the lung: a case report and literature review. Medicine..

[7] Maeda R, Isowa N, Onuma H, Miura H, Tokuyasu H, Kawasaki Y (2009). Primary malignant melanoma of the lung with rapid progression. Gen Thorac Cardiovasc Surg.

[8] Allen MS, Drash EC (1968). Primary melanoma of the lung. Cancer.

[9] Bagwell SP, Flynn SD, Cox PM, Davison JA (1989). Primary malignant melanoma of the lung. Am Rev Respir Dis.

[10] Ost D, Joseph C, Sogoloff H, Menezes G (1999). Primary pulmonary melanoma: case report and literature review. Mayo Clin Proc.

[11] Jensen OA, Egedorf J (1967). Primary malignant melanoma of the lung. Scand J Respir Dis.

[12] Postrzech-Adamczyk K, Chabowski M, Gluszczyk-Ferenc B, Wodzinska A, Muszczynska-Bernhard B, Szuba A (2015). Malignant melanoma of the lung: case series. Kardiochir Torakochirurgia Pol.

[13] Cota C, Saggini A, Lora V, Kutzner H, Rutten A, Sangueza O, et al.Uncommon Histopathological variants of malignant melanoma: part 1. Am J Dermatopathol. 2019;41:243–63. 10.1097/DAD.0000000000001218 30024414

[14] Alghanem AA, Mehan J, Hassan AA (1987). Primary malignant melanoma of the lung. J Surg Oncol.

[15] Lazarou I, Purek L, Duc C, Licker MJ, Spiliopoulos A, Tschopp JM (2014). Primary malignant achromic melanoma of the lung. Thoracic cancer.

[16] Liu GH, Liu J, Dong H, Tang XJ (2014). Primary malignant melanoma of the lung: a case report. Int J Clin Exp Med.

[17] Kundranda MN, Clark CT, Chaudhry AA, Chan V, Daw HA (2006). Primary malignant melanoma of the lung: a case report and review of the literature. Clin Lung Cancer.

[18] Yamamoto Y, Kodama K, Maniwa T, Takeda M, Tanaka Y, Ozawa K (2017). Primary malignant melanoma of the lung: a case report. Mol Clin Oncol.

[19] Yabuki H, Kuwana K, Minowa M (2018). Resection of primary malignant lung melanoma: a case report. Asian Cardiovasc Thorac Ann.

[20] Mahowald MK, Aswad BI, Okereke IC, Ng T (2015). Long-term survival after pneumonectomy for primary pulmonary malignant melanoma. Ann Thorac Surg.

[21] Yunce M, Selinger S, Krimsky W, Harley DP (2018). Primary malignant melanoma of the lung: a case report of a rare tumor and review of the literature. J Community Hosp Intern Med Perspect.

